# Clinical characteristics and all-cause mortality in female patients with idiopathic pulmonary fibrosis in Chinese population

**DOI:** 10.7189/jogh.15.04246

**Published:** 2025-08-29

**Authors:** Aiyuan Zhou, Qing Song, Rongli Lu, Dingding Deng, Yi Li, Xiyan Zhang, Pinhua Pan

**Affiliations:** 1 Department of Respiratory Medicine, National Key Clinical Specialty, Branch of National Clinical Research Centre for Respiratory Disease, Xiangya Hospital, Central South University, Changsha, Hunan, China; 2 Xiangya Hospital, Central South University, Centre of Respiratory Medicine, Changsha, Hunan, China; 3 Clinical Research Centre for Respiratory Diseases in Hunan Province, Changsha, Hunan, China; 4 Hunan Engineering Research Centre for Intelligent Diagnosis and Treatment of Respiratory Disease, Changsha, Hunan, China; 5 Xiangya Hospital, National Clinical Research Centre for Geriatric Disorders, Changsha, Hunan, China; 6 Department of Respiratory and Critical Care Medicine, The Second Xiangya Hospital, Central South University, Changsha, Hunan, China; 7 Department of Respiratory and Critical Care Medicine, First Affiliated People's Hospital of Shaoyang University, Shaoyang, Hunan, China

## Abstract

**Background:**

Idiopathic pulmonary fibrosis (IPF) exhibits notable sex-based disparities and male patients with IPF are significantly more numerous than female patients. Therefore, the female patients are often overlooked and under-investigated. This study aimed to evaluate the clinical characteristics and all-cause mortality in female patients with IPF in Chinese population.

**Methods:**

This retrospective cohort study included IPF patients registered at Xiangya Hospital of Central South University and the First Affiliated People's Hospital of Shaoyang University from January 2015 to May 2024. The data on age, sex, body mass index, smoking (pack-years), forced expiratory volume in one second to forced vital capacity, diffusing capacity of the lung for carbon monoxide percentage of predicted (DL_CO_ %pred), diffusing capacity divided by the alveolar volume (DL_CO_/VA), laboratory analysis, comorbidities, and antifibrotic therapy were collected. The patients were followed-up to collect the data on all-cause mortality.

**Results:**

A total of 583 patients were enrolled and 116 (19.9%) of them were female. Female patients had higher levels of forced expiratory volume in one second to forced vital capacity, DL_CO_ %pred, DL_CO_/VA, arterial oxygen partial pressure (Pao_2_), and total cholesterol, while a lower smoking (pack-years), haemoglobin, blood urea, uric acid, myoglobin, and creatine kinase. The proportion of lung cancer and antifibrotic therapy were lower in female patients (*P* < 0.05). Logistic regression analysis showed that haemoglobin (odds ratio (OR) = 0.813; 95% confidence interval (CI) = 0.706–0.937) and blood urea (OR = 0.158; 95% CI = 0.030–0.849) as negatively associated with female, while total cholesterol (OR = 14.699; 95% CI = 1.892–114.190) and DL_CO_ %pred (OR = 1.112; 95% CI = 1.005–1.229) were positively associated (*P* < 0.05). Over a median follow-up period of 31.0 (12.0–64.0) months, a total of 489 patients with IPF were analysed the all-cause mortality and 101 (20.7%) of them were female. Cox regression analysis revealed that female patients had significantly lower all-cause mortality compared to males (hazard ratio = 0.168; 95% CI = 0.031–0.920, *P* < 0.05).

**Conclusions:**

Significant differences in clinical characteristics and prognosis were observed between male and female IPF patients in Chinese population. Specifically, female patients exhibited better pulmonary function, higher Pao_2_, and lower all-cause mortality than male patients. Therefore, gender differences should be systematically evaluated in the diagnostic and therapeutic approach to IPF, and targeted strategies should be developed to optimise treatment outcomes in female patients.

Idiopathic pulmonary fibrosis (IPF) is a chronic, progressive, and destructive interstitial lung disease of unknown ethology, characterised by progressive dyspnoea and a significant decline in lung compliance. Characterised by high mortality and poor prognosis, IPF has a median survival time of 2.5–3.5 years following diagnosis [1,2]. As such, effective prevention and treatment strategies for this disease are urgently needed.

While environmental factors such as smoking, occupational and organic inhalants, and air pollution have been implicated in the development of IPF [3], there are also notable sex-related differences in the disease. Patients with IPF show a clear male predominance, whereas females are more likely to develop connective tissue disease-associated interstitial lung disease [4]. Previous studies have indicated that females represent only 30% of IPF patients [5]. A study by Sesé et al. [6] reported that females constituted 22% of IPF patients, with a significantly better forced vital capacity percentage of predicted (FVC %pred) compared to males, although no significant difference in survival between the sexes was observed. Female patients with IPF are often under-recognised due to the lower prevalence in this group. In fact, there were significant regional and ethnic differences among patients with IPF [7]. The smoking rate is relatively high among men in Europe and America (75% of IPF patients have a history of smoking), while indoor air pollution caused by the use of traditional fuels in rural areas of China is an important risk factor for the occurrence of IPF [8]. Additionally, there is limited epidemiological data about the prevalence of IPF in China, and there are few studies focusing on female IPF patients.

Therefore, the aim of this study was to investigate the clinical characteristics and all-cause mortality in female IPF patients from two large tertiary-grade A hospitals in China, in order to reveal some novel risk factors related to the pathogenesis of IPF and inform better prevention and treatment approaches for this disease.

## METHODS

### Study design and participants

This was a retrospective cohort study conducted at Xiangya Hospital of Central South University and the First Affiliated People's Hospital of Shaoyang University between January 2015–May 2024. The study included patients diagnosed with IPF based on the diagnostic criteria outlined in the official clinical practice guidelines for IPF established by the American Thoracic Society, European Respiratory Society, Japanese Respiratory Society, and Asociación Latinoamericana de Tórax (ATS/ERS/JRS/ALAT) [9,10].

The study was performed in compliance with the Declaration of Helsinki and was approved by the Ethics Committee of Xiangya Hospital of Central South University (Approval Number: 202406127). Informed consent was exempted due to the retrospective design of the study, and the analysis was performed using anonymised clinical data.

### Data collection and study procedures

All data were collected at the time of patients' initial hospital visits. Demographic information, including age, sex, body mass index (BMI), smoking history (pack-years), forced expiratory volume in one second percentage of predicted (FEV_1_%pred), forced vital capacity percentage of predicted (FVC %pred), FEV_1_/FVC ratio, diffusing capacity of the lung for carbon monoxide percentage of predicted (DL_CO_ %pred), diffusing capacity divided by alveolar volume (DL_CO_/VA), and laboratory test results, were recorded. Comorbidities such as hypertension, diabetes, chronic heart disease, lung cancer, chronic obstructive pulmonary disease (COPD), and obstructive sleep apnoea were also documented. Data on antifibrotic therapies, including pirfenidone, nintedanib, and lung transplantation, were collected. Patients were followed up to assess all-cause mortality. Based on sex, patients were categorised into male and female groups for analysis in this study.

### Sample size calculated

We used PASS software, version 15.0.5 (NCSS, LLC, Kaysville, UT, USA) to calculate the sample size. According to a previous study in Hunan province [11], the prevalence of the female patients with IPF was 28.5%. a confidence level of 0.95, and a confidence interval (CI) width (two-sided) of 0.05. Considering a dropout rate 20%, the minimum sample size was 392.

### Statistical analysis

Continuous variables that exhibited homogeneity of variance and a normal distribution were analysed using the *t* test and are presented as means with standard deviation (SD). For variables that did not meet these assumptions, non-parametric tests were employed, and results are expressed as medians with interquartile ranges (IQR). Categorical variables were analysed using the χ^2^ test. Logistic regression identified factors associated with female IPF patients using variables with *P*-values <0.05. To address collinearity between haemoglobin and red blood cell, and between DL_CO_ %pred and DL_CO_/VA, haemoglobin and DL_CO_ %pred were selected for the model (**Table 1**), with no observed multicollinearity (Table S1 in the **Online Supplementary Document**). Odds ratios (OR) with 95% CI were calculated. Multivariate Cox regression analysis identified factors associated with mortality using variables with *P*-values <0.05 (Table S2 in the **Online Supplementary Document**), with no multicollinearity (Table S3 in the **Online Supplementary Document**). Survival curves were generated for male-female group comparisons. A *P*-value <0.05 was considered statistically significant. Statistical analyses were conducted using SPSS version 26.0 (IBM, Armonk, New York, USA) and Free Statistics software version 1.7.1 (Beijing, China).

**Table 1:** All-cause mortality in female patients with IPF during follow-up

Variables	Total (n = 489)	Male (n = 388)	Female (n = 101)	*P-*value
All-cause mortality, n (%)				0.007*
*Yes*	140 (28.6)	122 (31.4)	18 (17.8)	
*No*	349 (71.4)	266 (68.6)	83 (82.2)	
Follow-up times (months), MD (IQR)	31.0 (12.0–64.0)	29.0 (11.8–64.0)	35.0 (16.0–69.0)	0.095

IPF – idiopathic pulmonary fibrosis, IQR – Interquartile range, MD – median

*P-value indicate statistical significance.

## RESULTS

### Clinical characteristics of female patients with IPF

**Figure 1:**
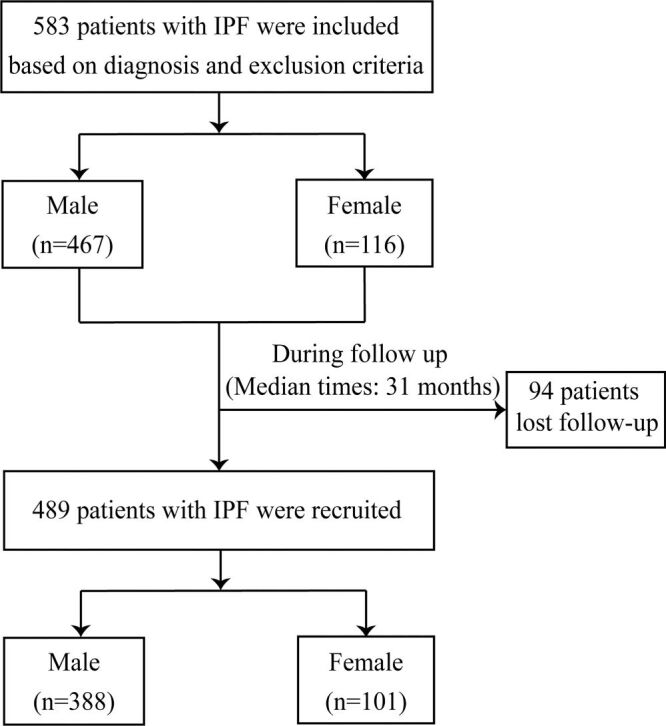
Flowchart of this study. IPF – idiopathic pulmonary fibrosis.

A total of 583 patients with IPF were included in this study (**Figure 1**). Among them, 116 (19.9%) were female. The mean age of the cohort was 66.2 (SD = 9.7) years (Table S4 in the **Online Supplementary Document**).

Female IPF patients exhibited higher values for FEV_1_/FVC (mean (x̄) = 84.8, SD = 6.7 *vs*. x̄ = 82.1, SD = 8.0; *P* = 0.008), DL_CO_ %pred (x̄ = 56.5, SD = 18.1 *vs*. x̄ = 46.8, SD = 17.5; *P* = 0.010), DL_CO_/VA (x̄ = 1.2, SD = 0.4 *vs*. x̄ = 1.0, SD = 0.3; *P* < 0.001), arterial oxygen partial pressure (Pao_2_) (x̄ = 90.7, SD = 25.1 *vs*. x̄ = 82.6, SD = 22.4; *P* < 0.001), total cholesterol (median (MD) = 4.6, IQR = 3.9–5.9 *vs*. MD = 4.1, IQR = 3.5–4.7; *P* < 0.001), and high-density lipoprotein cholesterol (MD = 1.3, IQR = 1.0–1.6 *vs*. MD = 1.0, IQR = 0.8–1.2; *P* < 0.001). Conversely, female patients had lower levels of smoking history (MD = 0, IQR = 0–0 *vs*. MD = 30, IQR = 0.1–47.5; *P* < 0.001), haemoglobin (x̄ = 123.2, SD = 18.4 *vs*. x̄ = 133.0, SD = 16.8; *P* < 0.001), red blood cell count (x̄ = 4.0, SD = 0.5 *vs*. x̄ = 4.3, SD = 0.6; *P* < 0.001), blood urea (x̄ = 4.9, SD = 1.5 *vs*. x̄ = 6.0, SD = 3.3; *P* < 0.001), uric acid (x̄ = 286.4, SD = 84.3 *vs*. x̄ = 344.8, SD = 94.7; *P* < 0.001), myoglobin (MD = 40.2, IQR = 31.4–51.7 *vs*. MD = 48.2, IQR = 36.6–66.2; *P* < 0.001), and creatine kinase (MD = 53.5, IQR = 39.4–74.2 *vs*. MD = 64.1, IQR = 42.6–93.5; *P* = 0.011). Additionally, the proportion of patients with lung cancer (0 *vs*. 5.8%; *P* = 0.008) and those receiving antifibrotic therapy was lower in the female group (48.3 *vs*. 61.9%; *P* = 0.008) (**Table 2**).

**Table 2:** Clinical characteristics of female patients with IPF

Variables	Male (n = 467)	Female (n = 116)	*P*-value
Age (years), x̄ ± SD	66.2 ± 9.8	66.0 ± 9.4	0.801
BMI (kg/m^2^), x̄ ± SD	24.4 ± 3.2	23.8 ± 4.1	0.141
Smoking (pack-years), MD (IQR)	30 (0.1–47.5)	0 (0–0)	<0.001*
FEV_1_%pred, x̄ ± SD	83.0 ± 20.3	83.4 ± 23.9	0.875
FVC %pred, x̄ ± SD	80.4 ± 22.1	79.1 ± 24.8	0.658
FEV_1_/FVC (%), x̄ ± SD	82.1 ± 8.0	84.8 ± 6.7	0.008*
DL_CO_ %pred, x̄ ± SD	46.8 ± 17.5	56.5 ± 18.1	0.010*
DL_CO_/VA (mmol/min/kPa/L), x̄ ± SD	1.0 ± 0.3	1.2 ± 0.4	<0.001*
Laboratory analysis			
*Lactic acid (mmol/L), MD (IQR)*	1.4 (1.1–1.8)	1.3 (0.9–1.8)	0.137
*Paco_2_ (mmHg), x̄ ± SD*	41.1 ± 6.1	42.0 ± 6.2	0.177
*Pao_2_ (mmHg), x̄ ± SD*	82.6 ± 22.4	90.7 ± 25.1	0.001*
*White blood cell (10^9^/L), x̄ ± SD*	7.6 ± 2.8	7.2 ± 2.3	0.160
*Haemoglobin (g/L), x̄ ± SD*	133.0 ± 16.8	123.2 ± 18.4	<0.001*
*Red blood cell (10^12^/L), x̄ ± SD*	4.3 ± 0.6	4.0 ± 0.5	<0.001*
*Blood platelet (10^9^/L), x̄ ± SD*	210.1 ± 85.0	220.4 ± 68.5	0.226
*Blood urea (mmol/L), x̄ ± SD*	6.0 ± 3.3	4.9 ± 1.5	<0.001*
*Uric Acid (μmol/L), x̄ ± SD*	344.8 ± 94.7	286.4 ± 84.3	<0.001*
*Lactic dehydrogenase (U/L), MD (IQR)*	240.0 (203.0–281.0)	234.5 (205.1–293.0)	0.861
*Myoglobin (μg/L), MD (IQR)*	48.2 (36.6–66.2)	40.2 (31.4–51.7)	<0.001*
*CK (U/L), MD (IQR)*	64.1 (42.6–93.5)	53.5 (39.4–74.2)	0.011*
*CK-MB (U/L), MD (IQR)*	13.9 (10.9–17.1)	13.5 (10.8–17.3)	0.793
*Total cholesterol (mmol/L), MD (IQR)*	4.1 (3.5–4.7)	4.6 (3.9–5.9)	<0.001*
*High-density lipoprotein cholesterol (mmol/L), MD (IQR)*	1.0 (0.8–1.2)	1.3 (1.0–1.6)	<0.001*
*Low-density lipoprotein cholesterol (mmol/L), MD (IQR)*	2.6 (2.1–3.1)	2.8 (2.3–3.5)	0.059
*Fibrinogen (g/L), MD (IQR)*	3.6 (2.9–4.4)	3.4 (2.8–4.1)	0.137
*Total bile acid (μmol/L), MD (IQR)*	4.7 (3.0–8.2)	4.4 (3.0–7.5)	0.602
*KL-6 (U/mL), MD (IQR)*	1158.0 (677.1–1908.0)	1240.0 (628.6–2284.0)	0.657
*Erythrocyte sedimentation rate (mm/h), MD (IQR)*	28.0 (13.0–55.0)	33 (17.8–46.0)	0.255
*C3 (mg/L), MD (IQR)*	0.9 (0.8–1.1)	0.9 (0.8–1.0)	0.299
*C4 (mg/L), MD (IQR)*	0.2 (0.2–0.3)	0.2 (0.2–0.3)	0.563
Comorbidities, n (%)			
*Hypertension*	131 (28.1)	33 (28.4)	0.932
*Diabetes*	106 (22.7)	20 (17.2)	0.201
*Chronic heart disease*	103 (22.1)	23 (19.8)	0.602
*Lung cancer*	27 (5.8)	0 (0.0)	0.008*
*COPD*	47 (10.1)	5 (4.3)	0.052
*OSA*	20 (4.3)	6 (5.2)	0.678
Antifibrotic therapy†, n (%)			0.008*
*No*	178 (38.1)	61 (52.1)	
*Yes*	289 (61.9)	56 (48.3)	

BMI – body mass index, C3 – complement 3, C4 – complement 4, COPD – chronic obstructive pulmonary disease, CK – creatine kinase, CK-MB – creatine kinase-myocardial band, DL_CO_ – diffusing capacity of the lung for carbon monoxide, DL_CO_ %pred – diffusing capacity of the lung for carbon monoxide percentage of predicted, DL_CO_/VA – diffusing capacity divided by the alveolar volume, FEV_1_%pred – forced expiratory volume in one second percentage of predicted, FVC %pred – forced vital capacity percentage of predicted, IPF – idiopathic pulmonary fibrosis, IQR – interquartile range, KL-6 – Krebs von den Lungen-6, kPa – kilopascal, MD – median, mmHg – millimetres of mercury, OSA – obstructive sleep apnoea, Paco_2_ – arterial carbon dioxide partial pressure, Pao_2_ – arterial oxygen partial pressure, SD – standard deviation, x̄ – mean.

*P-value indicate statistical significance.

**†**Antifibrotic therapy including lung transplantation, pirfenidone and nintedanib.

### Factors related to female patients with IPF

Logistic regression analysis revealed that haemoglobin (OR = 0.813; 95% CI = 0.706–0.937, *P* = 0.004) and blood urea (OR = 0.158; 95% CI = 0.030–0.849, *P* = 0.031) were negatively associated with female patients, whereas total cholesterol (OR = 14.699; 95% CI = 1.892–114.190, *P* = 0.010) and DL_CO_ %pred (OR = 1.112; 95% CI = 1.005–1.229, *P* = 0.039) were positively associated with female patients (Table S5 in the **Online Supplementary Document**).

### All-cause mortality in female patients with IPF

A total of 489 patients with IPF were included in the analysis of all-cause mortality, of whom 101 (20.7%) were female. The median follow-up duration for the entire cohort was 31.0 months (IQR = 12.0–64.0). Female patients had a longer median follow-up duration of 35.0 months (IQR = 16.0–69.0), compared to 29.0 months (IQR = 11.8–64.0) for male patients. Despite this difference, female patients exhibited a significantly lower all-cause mortality rate than their male counterparts (17.8 *vs*. 31.4%; *P* = 0.007), with an overall mortality rate of 28.6% in the study population (**Table 1**).

### Multivariate analysis of factors associated with all-cause mortality in patients with IPF

Univariate analysis showed that sex, age, BMI, FEV_1_%pred, FVC %pred, DL_CO_ %pred, DL_CO_/VA, Pao_2_, blood urea, lactic dehydrogenase, fibrinogen, erythrocyte sedimentation rate, lung cancer, COPD and antifibrotic therapy were related factors for all-cause mortality in IPF patients (Table S2 in the **Online Supplementary Document**).

**Figure 2:**
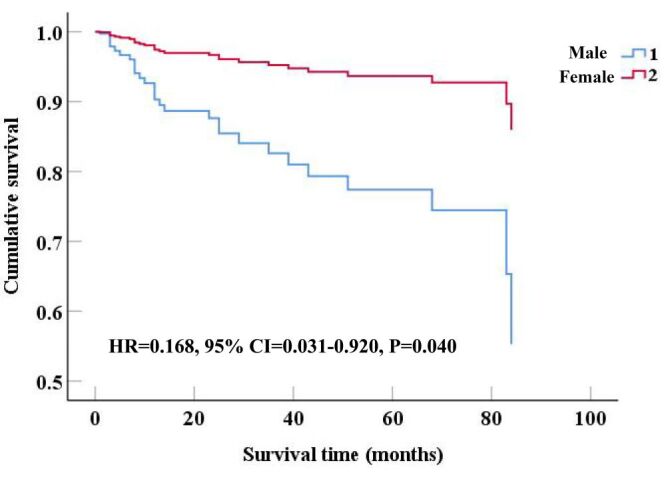
Cumulative survival of female and male patients with IPF during follow-up. A *P*-value <0.05 was considered statistically significant. CI – confidence interval, HR – hazard ratio, IPF – idiopathic pulmonary fibrosis.

After adjusting for confounding factors including BMI, FEV_1_%pred, FVC %pred, DL_CO_/VA, white blood cell, blood urea, fibrinogen, erythrocyte sedimentation rate, COPD and antifibrotic therapy, Cox regression analysis revealed that age (hazard ratio (HR) = 1.073; 95% CI = 1.016–1.133, *P* = 0.012), lactate dehydrogenase (HR = 1.006; 95% CI = 1.000–1.011, *P* = 0.040), and the comorbidity of lung cancer (HR = 3.964; 95% CI = 1.007–15.603, *P* = 0.049) were significantly associated with all-cause mortality (**Table 3**). In addition, the survival curve indicated that female patients had a lower all-cause mortality (HR = 0.168; 95% CI = 0.031–0.920, *P* = 0.040) (**Figure 2**).

**Table 3:** Cox regression analysis of factors associated with all-cause mortality in patients with IPF*

Variables	HR	95% CI	*P*-value
Sex			
*Male*	Reference		
*Female*	0.168	0.031–0.920	0.040†
Age (years)	1.073	1.016–1.133	0.012†
BMI (kg/m^2^)	1.041	0.896–1.209	0.601
FEV_1_%pred	0.950	0.890–1.014	0.121
FVC %pred	1.023	0.967–1.082	0.437
DL_CO_/VA (mmol/min/kPa/L)	0.329	0.072–1.505	0.152
White blood cell (10^9^/L)	1.058	0.838–1.336	0.634
Blood urea (mmol/L)	0.920	0.706–1.200	0.540
Lactic dehydrogenase (U/L)	1.006	1.000–1.011	0.040†
Fibrinogen (g/L)	1.219	0.781–1.903	0.383
Erythrocyte sedimentation rate (mm/h)	0.993	0.974–1.012	0.476
COPD	0.953	0.240–3.787	0.945
Lung cancer	3.964	1.007–15.603	0.049†
Antifibrotic therapy	2.205	0.704–6.904	0.174

BMI – body mass index, CI – confidence interval, COPD – chronic obstructive pulmonary disease, DL_CO_/VA – diffusing capacity divided by the alveolar volume, FEV_1_%pred – forced expiratory volume in one second percentage of predicted, FVC %pred – forced vital capacity percentage of predicted, HR – hazard ratio, IPF – idiopathic pulmonary fibrosis, kPa – kilopascal

*Variables in Cox regression model: sex, BMI, FEV_1_%pred, FVC %pred, DL_CO_/VA, blood urea, white blood cell, lactic dehydrogenase, fibrinogen, erythrocyte sedimentation rate, COPD, lung cancer and anti-fibrotic therapy.

†*P*-value indicate statistical significance.

## DISCUSSION

This retrospective cohort study demonstrated that female IPF patients exhibited better pulmonary function, a lower smoking history, and a reduced prevalence of lung cancer compared to males in Chinese population. After adjusting for confounding factors, survival analysis indicated a potentially lower all-cause mortality in female patients, which may reflect sex-related differences in disease progression and prognosis.

So far, only few studies have examined the clinical characteristics and all-cause mortality of IPF based on gender. Given the higher prevalence of IPF in males, female patients with IPF are often under recognised in clinical practice. However, understanding the clinical characteristics and prognosis of female patients is crucial for improving prevention and treatment strategies, particularly within the Chinese population. In this study, female patients accounted for 20% of the IPF cohort. Several studies have reported that the proportion of female IPF patients ranges from 20–30%, which is consistent with our findings [6,12]. Furthermore, it is noteworthy that the proportion of female smokers in this study was relatively small. Previous studies have also indicated that men had higher smoking exposure [6,13]. Given that smoking is a significant environmental risk factor for IPF [14,15], the low overall prevalence of smoking among women in China [16] may have influenced our findings.

In this study, we found that female IPF patients had better pulmonary function, characterised by higher FEV_1_/FVC, DL_CO_ % predicted, DL_CO_/VA, and Pao_2_. Similarly, Kalafatis et al. [12] reported that men with IPF had significantly lower FVC % predicted and total lung capacity % predicted than women. Consistent with our findings, Sesé et al. [6] observed higher baseline FVC % predicted in females than in males. Likewise, Zaman et al. [17] reported that women had higher baseline FVC % predicted and DL_CO_ % predicted.

Furthermore, for the first time, we demonstrated that female IPF patients had higher total cholesterol levels, while exhibiting lower levels of haemoglobin, red blood cell count, blood urea, uric acid, myoglobin, and creatine kinase. Previous studies have suggested that total cholesterol (TC) may serve as a biomarker for predicting mortality in patients with acute exacerbation of idiopathic pulmonary fibrosis (AE-IPF) [18], with another study identifying lower TC levels as an independent risk factor for mortality [19]. Our finding that TC levels are higher in female IPF patients compared to males may further support the hypothesis that female patients are less severely affected by the disease than their male counterparts. Additionally, the proportion of lung cancer was lower in female patients with IPF, which may be attributed to smoking being a major risk factor for lung cancer, with a higher prevalence of smoking in male patients [20]. We also noted that a lower proportion of female patients received antifibrotic therapy, which is primarily attributed to the generally milder symptoms observed in females. Given the high cost of antifibrotic medications, many patients are reluctant to use them unless they experience significant shortness of breath. Previous studies have confirmed that female patients with IPF are less likely to undergo lung transplantation [6]. Furthermore, we are the first to find that the haemoglobin, blood urea, total cholesterol and DL_CO_ %pred were independently associated with female patients. However, the OR of total cholesterol was high and this might be related to the bias value of the patients.

In this study, we found that female patients with IPF had a lower all-cause mortality. Furthermore, we identified several independent factors associated with all-cause mortality in IPF, including age, lactic dehydrogenase, and comorbid lung cancer. Although in a prospective cohort study, Sesé et al. [6] found no association between female sex and improved survival after adjusting for age and FVC. However, there was a small number of patients and a lack of power. In addition, a national registry study (1992–2003) reported the age-adjusted mortality rate increased 28.4% in male and 41.3% in female. The rate of increase was higher in female [21]. This is inconsistent with our results and this might be related to the regional differences. However, a retrospective observational study from Caminati et al. [22] found that females had better survival than males after adjusting for age and comorbidities. Moreover, a retrospective cohort study from Zaman et al. [17] identified male sex as an independent risk factor for higher mortality. Han et al. [23] further observed worse survival in males with IPF, a disparity that persisted after adjusting for exertional desaturation and FVC % pred. These were similar to our results.

We hypothesise that the male predominance in IPF may be related to sex hormones. This hypothesis is supported by findings in mice, which align with clinical observations showing a higher prevalence of pulmonary fibrosis in males than in females. One study demonstrated that male mice develop more severe bleomycin-induced pulmonary fibrosis than age-matched females [24]. Additionally, research suggests that airway fibrosis is influenced by both relaxin and oestrogen, with oestrogen providing a protective effect in the absence of relaxin [25]. In contrast, male sex hormones appear to exacerbate lung function deterioration following bleomycin-induced pulmonary fibrosis [26]. Oestrogen appears to exert anti-fibrotic effects potentially through multiple pathways. Experimental evidence demonstrates that 17β-oestradiol administration significantly attenuates Transforming Growth Factor Beta 1 (TGF-β1) expression in pulmonary tissues, while pharmacological oestrogen receptor antagonists conversely potentiate TGF-β1-driven fibrotic processes [27,28]. Mechanistically, 17β-oestradiol exerts anti-fibrotic effects through down-regulation of key fibrosis-associated proteins, including chloride intracellular channel 3 and retinol-binding protein 7 [29]. Furthermore, oestrogen deficiency induces pathological activation of the renin-angiotensin system, resulting in up-regulated expression of pro-fibrotic mediators and initiation of a self-perpetuating fibrotic cascade that ultimately promotes pulmonary fibrosis pathogenesis [30]. However, the impact of sex and sex hormones on respiratory regulation remains incompletely elucidated, and the role of sex hormones in the fibrotic process warrants further investigation.

This study also has several limitations. First, the proportion of female patients who smoke is relatively small, and it is necessary to expand the sample size in future studies to better assess this factor. Second, computed tomography features may hold significant clinical relevance, which represents a potential avenue for future research. Moreover, although the data set contains missing data for this parameter, we emphasise that it does not represent a central analytical target in this study and consequently has no bearing on the validity or interpretation of the principal findings. Finally, the mortality of CI for the HR is quite wide in female patients and this might be caused by the relatively small sample size, underscoring the need for multi-centre studies with larger sample sizes in the future.

## CONCLUSIONS

Significant differences in clinical characteristics and prognosis were observed between male and female IPF patients in Chinese population. Specifically, female patients exhibited better pulmonary function, higher Pao_2_, and lower all-cause mortality than male patients. Therefore, gender differences should be systematically evaluated in the diagnostic and therapeutic approach to IPF, and targeted strategies should be developed to optimise treatment outcomes in female patients.

## Additional material


Online Supplementary Document
