# Supplementary material for: Clinical characteristics and all-cause mortality in female patients with idiopathic pulmonary fibrosis in Chinese population
Source: J Glob Health. 2025 Aug 29;15:04246. doi: 10.7189/jogh.15.04246 (PMC12395208; doi:10.7189/jogh.15.04246)
Supplement: Online Supplementary Document [file jogh-15-04246-s001.pdf]

Supplement to: Zhou A, Song Q, Lu R, Deng D, Li Y, Zhang X, Pan P. Clinical characteristics and all-cause mortality in female patients with idiopathic pulmonary fibrosis in Chinese population. J Glob Health. 2025;15:04246.

Supplement Table 1. The multicollinearity was conducted of variables in logistic regression model

Variables	Tolerance	VIF
FEV_1/FVC (%)	0.862	1.160
DL_{CO} %pred	0.600	1.666
PaO_2 (mmHg)	0.795	1.257
Hemoglobin (g/L)	0.671	1.489
Blood urea (mmol/L)	0.693	1.443
Uric Acid (μmol/L)	0.434	2.305
Myoglobin (μg/L)	0.669	1.495
CK (U/L)	0.656	1.523
Total cholesterol (mmol/L)	0.715	1.399
High-density lipoprotein cholesterol (mmol/L)	0.651	1.537
Lung cancer	0.836	1.197

CK – creatine kinase, DL_{CO} %pred – diffusing capacity of the lung for carbon monoxide percentage of predicted, FEV_1 – forced expiratory volume in one second, FVC – forced vital capacity, mmHg – millimetres of mercury, PaO_2 – arterial oxygen partial pressure, VIF – variance inflation factor

Supplement Table 2. Univariate analysis of risk factors for all-cause mortality in patients with IPF

Variables	Univariate		
	**OR**	**95%CI**	***P*-value**
Sex			0.008*
Male	Reference		
Female	0.470	0.270–0.820	
Age (years)	1.003	1.010–1.050	0.013*
BMI (kg/m^2)	0.924	0.855–0.994	0.035*
Smoking (pack-years)	1.001	0.994–1.008	0.832
FEV_1 %pred	0.976	0.964–0.989	< 0.001*
FVC %pred	0.977	0.964–0.990	< 0.001*
FEV_1 /FVC (%)	1.000	0.969–1.033	0.982
DL_{CO} %pred	0.950	0.923–0.978	< 0.001*
DL_{CO}/VA (mmol/min/kPa/L)	0.131	0.051–0.338	< 0.001*
Lab analysis			
Lactic acid (mmol/L)	1.195	0.873–1.637	0.266
$PaCO_2$ (mmHg)	0.983	0.948–1.019	0.342
PaO_2 (mmHg)	0.993	0.984–1.003	0.174
White blood cell (10^9/L)	1.179	1.093–1.271	< 0.001*
Haemoglobin (g/L)	0.994	0.982–1.005	0.281
Red blood cell (10^{12}/L)	0.876	0.624–1.228	0.442
Blood platelet (10^9/L)	0.999	0.997–1.002	0.664
Blood urea (mmol/L)	1.081	1.007–1.160	0.032*
Uric Acid (μmol/L)	1.000	0.998–1.002	0.732
Lactic dehydrogenase (U/L)	1.004	1.002–1.007	< 0.001*
Myoglobin (μg/L)	1.002	0.999–1.005	0.137
CK (U/L)	0.999	0.997–1.002	0.634
CK-MB (U/L)	1.012	0.988–1.037	0.330
Total cholesterol (mmol/L)	0.795	0.629–1.003	0.053
High-density lipoprotein cholesterol (mmol/L)	0.579	0.286–1.173	0.129
Low-density lipoprotein cholesterol (mmol/L)	0.840	0.638–1.106	0.214
Fibrinogen (g/L)	1.357	1.145–1.609	< 0.001*
Total bile acid (μmol/L)	1.011	0.991–1.033	0.281
KL-6 (U/mL)	1.000	1.000–1.001	0.247
Erythrocyte sedimentation rate (mm/h)	1.018	1.011–1.025	< 0.001*
C3 (mg/L)	0.494	0.145–1.681	0.259

C4 (mg/L)	1.309	0.054–31.454	0.868
Comorbidities			
Hypertension	1.061	0.688–1.635	0.789
Diabetes	1.114	0.691–1.793	0.658
Chronic heart disease	1.005	0.619–1.631	0.984
Lung cancer	5.429	2.142–13.759	< 0.001*
COPD	2.023	1.106–3.700	0.022*
OSA	0.252	0.058–1.095	0.066
Antifibrotic therapy†			0.025*
No	Reference		
Yes	0.636	0.428–0.945	

BMI – body mass index, CI – confidence interval, C3 – complement 3, C4 – complement 4, COPD – chronic obstructive pulmonary disease, CK – creatine kinase, CK-MB – creatine kinase-myocardial band, DL_{CO} – diffusing capacity of the lung for carbon monoxide, DL_{CO} %pred – diffusing capacity of the lung for carbon monoxide percentage of predicted, DL_{CO}/VA – diffusing capacity divided by the alveolar volume, FEV_1 %pred – forced expiratory volume in one second percentage of predicted, FVC %pred – forced vital capacity percentage of predicted, IPF – idiopathic pulmonary fibrosis, IQR – interquartile range, KL-6 – Krebs von den Lungen-6, kPa – kilopascal, mmHg – millimetres of mercury, OR – odds ratio, OSA – obstructive sleep apnea, $PaCO_2$ – arterial carbon dioxide partial pressure, PaO_2 – arterial oxygen partial pressure, SD – standard deviation

**P*-value indicate statistical significance.

†Anti-fibrotic therapy including lung transplantation, pirfenidone and nidanib.

Supplement Table 3. The multicollinearity was conducted of variables in COX regression model

Variables	Tolerance	VIF
Sex	0.869	1.151
Age (years)	0.752	1.330
BMI (kg/m^2)	0.881	1.135
FEV_1 %pred	0.708	1.412
FVC %pred	0.718	1.393
DL_{CO}/VA (mmol/min/kPa/L)	0.799	1.252
White blood cell (10^9/L)	0.684	1.461
Blood urea (mmol/L)	0.853	1.173
Lactic dehydrogenase (U/L)	0.711	1.406
Fibrinogen (g/L)	0.562	1.780
Erythrocyte sedimentation rate (mm/h)	0.566	1.767
COPD	0.854	1.171
Lung cancer	0.909	1.100
Antifibrotic therapy	0.874	0.144

BMI – Body mass index, 95%CI – 95% Confidence interval, COPD – Chronic obstructive pulmonary disease, DL_{CO}/VA – Diffusing capacity divided by the alveolar volume, FEV_1 %pred – Forced expiratory volume in one second percentage of predicted, FVC %pred – Forced vital capacity percentage of predicted, IPF – Idiopathic pulmonary fibrosis, kPa – kilopascal, VIF – Variance inflation factor

Supplement Table 4. Baseline clinical characteristics of the total cohort of IPF patients

**Variables**	**Total (n = 583)**
Age (years), x̄ ± SD	66.2 ± 9.7
Sex, n (%)	
Male	467 (80.1)
Female	116 (19.9)
BMI (kg/m^2), x̄ ± SD	24.3 ± 3.4
Smoking (pack-years), MD (IQR)	20 (0–40)
FEV_1 %pred, x̄ ± SD	83.1 ± 21.1
FVC %pred, x̄ ± SD	80.1 ± 21.7
FEV_1/FVC (%), x̄ ± SD	82.7 ± 7.8
DL_{CO} (mmol/min/kPa), x̄ ± SD	3.6 ± 1.4
DL_{CO} %pred, x̄ ± SD	48.9 ± 18.0
DL_{CO}/VA (mmol/min/kPa/L), x̄ ± SD	1.1 ± 0.3
Lab analysis	
Lactic acid (mmol/L), MD (IQR)	1.4 (1.0–1.8)
$PaCO_2$ (mmHg), x̄ ± SD	41.3 ± 6.1
PaO_2 (mmHg), x̄ ± SD	84.3 ± 23.2
White blood cell (10^9/L), x̄ ± SD	7.5 ± 2.7
Haemoglobin (g/L), x̄ ± SD	131.0 ± 17.6
Red blood cell (10^{12}/L), x̄ ± SD	4.3 ± 0.6
Blood platelet (10^9/L), x̄ ± SD	212.2 ± 82.0
Blood urea (mmol/L), x̄ ± SD	5.7 ± 3.0
Uric Acid (μmol/L), x̄ ± SD	333.1 ± 95.6
Lactic dehydrogenase (U/L), MD (IQR)	239.8 (203.4–282.0)
Myoglobin (μg/L), MD (IQR)	46.0 (35.8–62.0)
CK (U/L), MD (IQR)	61.7 (41.4–90.6)
CK-MB (U/L), MD (IQR)	13.8 (10.9–17.1)
Total cholesterol (mmol/L), MD (IQR)	4.2 (3.6–4.9)
High-density lipoprotein cholesterol (mmol/L), MD (IQR)	1.0 (0.8–1.2)
Low-density lipoprotein cholesterol (mmol/L), MD (IQR)	2.6 (2.1–3.2)
Fibrinogen (g/L), MD (IQR)	3.5 (2.9–4.4)
Total bile acid (μmol/L), MD (IQR)	4.7 (3.0–8.1)
KL-6 (U/mL), MD (IQR)	1163.0 (670.9–1974.0)
Erythrocyte sedimentation rate (mm/h), MD (IQR)	28.0 (13.0–53.8)
C3 (mg/L), MD (IQR)	0.9 (0.8–1.1)

C4 (mg/L), MD (IQR)	0.2 (0.2–0.3)
Comorbidities, n (%)	
Hypertension	164 (28.1)
Diabetes	126 (21.6)
Chronic heart disease	126 (21.6)
Lung cancer	27 (4.6)
COPD	52 (8.9)
OSA	26 (4.5)
Anti-fibrotic therapy*, n (%)	
No	239 (40.8)
Yes	345 (59.2)

BMI – body mass index, C3 – complement 3, C4 – complement 4, COPD – chronic obstructive pulmonary disease, CK – creatine kinase, CK-MB – creatine kinase-myocardial band, DL_{CO} – diffusing capacity of the lung for carbon monoxide, DL_{CO} %pred – diffusing capacity of the lung for carbon monoxide percentage of predicted, DL_{CO}/VA – diffusing capacity divided by the alveolar volume, FEV_1 %pred – forced expiratory volume in one second percentage of predicted, FVC %pred – forced vital capacity percentage of predicted, IPF – idiopathic pulmonary fibrosis, IQR – interquartile range, KL-6 – Krebs von den Lungen-6, kPa – kilopascal, MD – median, mmHg – millimetres of mercury, OSA – obstructive sleep apnoea, $PaCO_2$ – arterial carbon dioxide partial pressure, PaO_2 – arterial oxygen partial pressure, SD – standard deviation, $\bar{x}$ – mean

***Anti-fibrotic therapy including lung transplantation, pirfenidone and nintedanib.

Supplement Table 5. Multivariate analysis of independent factors associated with female patients with IPF*

**Variables**	**OR**	**95% CI**	***P*-value**
Haemoglobin (g/L)	0.813	0.706–0.937	0.004†
DL_{CO} %pred	1.112	1.005–1.229	0.039†
Total cholesterol (mmol/L)	14.699	1.892–114.190	0.010†
Blood urea (mmol/L)	0.158	0.030–0.849	0.031†

CI – 95% confidence interval, CK – creatine kinase, DL_{CO} %pred – diffusing capacity of the lung for carbon monoxide percentage of predicted, DL_{CO}/VA – diffusing capacity divided by the alveolar volume, FEV_1/FVC – forced expiratory volume in 1 second /forced vital capacity ratio, IPF – idiopathic pulmonary fibrosis, OR – odds ratio, PaO_2 – arterial oxygen partial pressure

*Variables in logistic regression model: FEV_1/FVC, DL_{CO}/VA, DL_{CO} %pred, PaO_2, haemoglobin, red blood cell, uric acid, blood urea, myoglobin, CK, total cholesterol, high-density lipoprotein, and lung cancer.

†*P*-value indicate statistical significance.
